# α-Synuclein Seed Amplification Status Modifies the Relationship Between Plasma p-tau217, Amyloid and Fibrillar Tau

**DOI:** 10.21203/rs.3.rs-11271891/v1

**Published:** 2026-10-22

**Authors:** Íñigo Rodríguez-Baz, Rosa Ferrer, Marc Sans Falip, Elena Vera-Campuzano, Isabel Sala, Lucía Maure-Blesa, Judit Selma-González, Jesús García-Castro, Ignacio Illán Gala, Ana Bueno, Lídia Vaqué-Alcázar, Maria Franquesa-Mullerat, Alexandre Bejanin, Pablo Zaragoza-Ballester, Valle Camacho, Juan Fortea, Alberto Lleò, Mireia Tondo, Carla Abdelnour, Daniel Alcolea

## Abstract

Background. Plasma p-tau217 is sufficient to establish a biological diagnosis of Alzheimer's disease (AD) and is and is increasingly used as a routine diagnostic tool. Many patients with AD also carry α-synuclein pathology, detectable by cerebrospinal fluid (CSF) seed amplification assay (αSynSAA). Seeding activity has been linked to more fibrillar tau than amyloid burden alone would predict, but whether this influences plasma p-tau217 levels, or compromises classification at clinical thresholds, is unknown. Methods. Cross-sectional analysis of the Alzheimer's Disease Neuroimaging Initiative. Plasma p-tau217 (Fujirebio Lumipulse) was compared by αSynSAA status conditional on amyloid PET Centiloids, the CSF p-tau181/Aβ42 ratio and tau PET, using generalised additive models adjusted for age, sex, *APOE* ε4 status and cognitive stage (n = 193, of whom 33 αSynSAA-positive; CSF-referenced n = 286; amyloid PET-referenced n = 246). We quantified the attenuation contributed by each reference measure, modelled fibrillar tau against amyloid and against plasma p-tau217 by seeding status, and reclassified αSynSAA-positive participants after removing the estimated seeding-associated increment. Results. αSynSAA-positive participants had higher plasma p-tau217 levels at a given amyloid PET burden (27.3%, 95% CI 4.9–54.5; p = 0.015) and CSF ratio (22.9%, 1.7–48.7; p = 0.033), but not at a given tau PET burden (12.1%, − 10.3–40.3; p = 0.313); tau PET attenuated the association by 55.4%, more than the CSF ratio (31.1%) or amyloid PET (25.4%). The amyloid–tau relationship was steeper in αSynSAA-positive participants (p = 0.047), yet a given plasma p-tau217 level corresponded to the same fibrillar tau burden in both groups (2.5% higher, − 2.7–8.0; p = 0.347). Removing the increment did not improve agreement with either reference standard and modestly worsened it (accuracy 93.9% to 92.4% against the CSF ratio; 84.6% to 82.7% against amyloid PET), with the loss falling on sensitivity. Conclusions. Plasma p-tau217 is elevated in αSynSAA-positive individuals at equivalent AD pathology measured by amyloid PET or the CSF ratio, and this elevation is attenuated most by fibrillar tau. Neither analysis designed to detect artefactual inflation found evidence of it. A plasma p-tau217 result should not be down-weighted in patients with concomitant Lewy-related pathology.

